# The impact of excess body weight at the hospital frontline

**DOI:** 10.1186/1741-7015-12-64

**Published:** 2014-04-17

**Authors:** Andrew G Renehan, Iain E Buchan

**Affiliations:** 1 MRC Health eResearch Centre, Farr Institute for Health Informatics Research, University of Manchester, Manchester, UK; 2 Institute of Cancer Sciences, University of Manchester, Manchester, UK; 3 Department of Surgery, University of Manchester, Manchester Academic Health Science Centrem The Christie NHS Foundation Trust, Wilmslow Road, Manchester M20 4BX, UK

**Keywords:** Body mass index, Hospital admission rates, Data linkage

## Abstract

Quantification of disease burden by deaths or years lived with disability is a useful indicator as it informs prevention by accounting for health loss but it does not reflect the needs for health services. An alternative indicator is to quantify the impact of a risk factor on health care utilization. In an article published in *BMC Medicine*, Reeves and colleagues describe the relationship between body mass index in 1.2 million women (England) and hospital admission rates. The main finding was that around one in eight hospital admissions was attributable to overweight or obesity, translating to around 420,000 extra hospital admissions, and two million extra days spent in hospital, annually. These findings reinforce the evidence that excess body weight is associated with extensive healthcare utilization and emphasize the need to scale-up and speed-up research if global problems, such as obesity, are to be tackled with due alacrity.

Please see related research: http://www.biomedcentral.com/1741-7015/12/45.

## Background

Excess body weight, commonly measured as body mass index (BMI) ≥25 kg/m^2^ (overweight) and BMI ≥30 (obese), is an established risk factor for mortality and morbidity from cardiovascular disease [1], type 2 diabetes [2], cancer [3] and osteoarthritis [4]. An estimated three million deaths per year worldwide are attributed to excess body weight [5]. The Global Burden of Disease project [6] ranked excess body weight as the sixth largest cause of death and disability-adjusted life years (DALYs; sum of years lived with disability and years of life lost) after considering the independent effects of 67 different risk factors, clustered for 21 regions in the year 2010. This ranking increased from seventh in 1990 and was mainly accounted for by obesity/overweight-related cardiovascular disease, diabetes, cancer and musculoskeletal disorders [6]. Quantification of the disease burden by deaths or DALYs is useful as it informs prevention by accounting for health loss but it does not reflect the needs for health services. An alternative indicator is to quantify the impact of a risk factor, in this example, excess body weight, on the utilization of health care services.

### BMI and hospital utilization (England)

In an article published in *BMC Medicine*, Reeves and colleagues describe the relationship between BMI, determined at baseline (1996 to 2001) in the Million Women Study (age 50 to 64 years) and hospital admission rates over a 9.2 year follow-up period [7]. The main finding was, that among these women in England, around one in eight hospital admissions was attributable to overweight or obesity, translating to around 420,000 extra hospital admissions, and two million extra days spent in hospital, annually. The authors examined 25 types of indications for admission – of these, significant increases in the risk of admission with increasing BMI were observed for 19. Almost two-thirds (62% of first time admission) were for diabetes, ischemic heart disease, stroke, joint replacements, gallbladder disease or cancer.

To clinicians at the hospital frontline, these figures come as no surprise and are readily believed. By the nature of the study, the analysis is limited to women; therefore, the numbers of hospital admissions attributed to excess body weight for the general population may be twice the reported rates. The hospital admission data covered a period up to the end of 2008 – so, as trends of median BMI values in England have continued to increase since then (albeit with some slowing down) [8], current admission rates attributed to excess body weight may be higher.

The impressive numbers (over 1.2 million women with BMI measurements) included in this study allowed for powerful analyses, not only in the full group of women but also in subgroups defined by ten age bands after age 50 years. The near parallel curves for the relationships of BMI with rates of hospital admission suggest no interaction between age and BMI; in other words, the excess numbers of hospitalizations attributable to overweight and obesity impact similarly in middle and older ages. Given that much of the obesity epidemic in England is driven by weight gain in early adulthood (‘the fat getting fatter’) [8], these relationships make grim reading for those managing the already overstretched health care systems [9].

A particularly useful insight from this study is that a third of first admissions in the overweight and obese women were due mainly to venous thromboembolism, diverticular disease, diaphragmatic hernia, cataracts or carpal tunnel syndrome. These observations stretch the usual radar beyond common conditions causally associated with BMI, such as diabetes, thereby giving a fuller reflection of the impacts of excess body weight. Consider Saint’s triad of diverticular disease, hiatus hernia and gallbladder disease, traditionally thought to have no common pathophysiology and used as a counterexample to the commonly used ‘single cause of disease’ principle in diagnostic medicine of Occam's Razor [10]. Here, obesity may be the common pathophysiological mechanism and large datasets will allow one to test the hypothesis that these overlapping conditions are explained by one pathway.

### Several avenues for more investigation

The study by Reeves and colleagues confirms and extends the evidence that a high burden of hospital admissions in the United Kingdom is attributable to excess bodyweight [11,12]. This adds to the need for policy-makers to prioritize anti-obesity strategies, but also raises questions about entry points for intervention. The words of British epidemiologist, William Farr (1807 to 1883) ‘Diseases are more easily prevented than cured and the first step to their prevention is the discovery of their exciting causes’ are germane to this situation. The current study illuminates several avenues for more investigation:

First, there are opportunities to use hospital admissions as an outcome measure to assess population-level interventions. For example, hospital admissions rates are likely to reflect the effects of public health interventions sooner than mortality and with greater relevance than the usual process measures. This was clearly the case in tobacco control when demonstrating significant reductions in acute admissions for myocardial infarction following the introduction of the smoking bans in Scotland [13] and Liverpool [14].

Second, there is a need to distinguish unplanned from planned admissions, on which anti-obesity programs might have differential impacts. Third, it is unlikely that excess body weight, as a risk factor, and hospital admission rates, are causal in isolation. For example, data from two Scottish populations show that excess BMI is associated with a 30% increase in liver disease mortality, which is dwarfed by the observation of a 300% increase associated with excess alcohol consumption [15]. However, when these two risk factors occur together, there is a supra-additive interaction, and a 900% increase in liver disease mortality.

Fourth, against these complexities, there is a need to evaluate the cost of excess hospital admission attributable to excess weight. Withrow and Alter [16] estimated that obesity accounts for between 0.7% and 2.8% of world country's total healthcare expenditures, while the FORESIGHT project forecasted that billions of pounds will be consumed in 2020 due to obesity [17]. However, these models do not directly include hospital admission costs, and for example, they do not rank these costs against those attributable to smoking, alcohol or trauma. Such data are required for health resource allocation decisions at a governmental level.

Patients and the public are best served if such research is done in a timely manner using all relevant data. In the UK, there are particularly strong primary care data which may be linked with hospital admissions and used to adjust for many potential confounding factors. This work can be challenging in terms of data acquisition, information extraction, cleaning and analysis, requiring a concert of informatics, statistics and epidemiology. To harness such data for research, the UK recently established the Farr Institute for Health Informatics Research [18] creating a network of expertise alongside the physical and electronic infrastructure required to pursue research questions with linked health datasets at the national scale, quickly. Initiatives like the Farr Institute will need to be developed and linked internationally if policies about global public health problems, such as obesity, are to be informed properly.

## Conclusions

The findings of Reeves and colleagues reinforce the evidence that excess body weight is associated with extensive healthcare utilization. Similar signals may have languished for many years across other data sources. It is time to consider scaling-up and speeding-up research with linked data if global problems, such as obesity, are to be tackled with due alacrity.

## Competing interests

The authors declare they have no competing interests.

## Authors’ contributions

Both authors contributed to conception of the article. AGR drafted the article. Both authors were involved in editing and revision of the manuscript and both agreed to its publication.
